# Determination of optimal sampling wavelengths for reflectance measurement using sparse principal component analysis

**DOI:** 10.1038/s41598-026-49066-1

**Published:** 2026-04-18

**Authors:** Seyed Ali Amirshahi, Seyed Hossein Amirshahi, Hengameh Shirzadi

**Affiliations:** 1 Department of Computer Science, Norwegian University of Science and Technology, Teknologivegen 22, Gjøvik, 2815 Norway; 2 Department of Textile Engineering, Amirkabir University of Technology, Tehran Polytechnic, No. 350, Hafez Ave, Valiasr Square, Tehran, 1591634311 Iran

**Keywords:** Applied optics, Optical physics, Spectrophotometry

## Abstract

In the visible region, material reflectance spectra are typically recorded over the 400–700 nm range at uniformly spaced wavelength intervals (e.g., 1, 5, 10, or 20 nm). However, such uniform sampling strategies neglect the intrinsic spectral characteristics of materials and may fail to capture critical spectral features, particularly when the number of measurement channels are limited. In this study, Sparse Principal Component Analysis (SPCA) was employed to determine the most informative wavelengths for reflectance acquisition when the number of available spectral bands was constrained to 16, 13, 11, 9, and 7. The selected wavelengths were assigned as the central positions of Gaussian filters with very narrow bandwidths (FWHM = 1 nm). These filters were subsequently used to measure the reflectance spectra of 1269 Munsell color chips at a 1-nm spectral resolution. For comparison, reflectance measurements were also simulated using the same Gaussian filters, but with uniformly distributed central wavelengths across the visible spectrum. The reconstruction performances of both approaches were quantitatively evaluated using the Root Mean Square Error (RMSE), Goodness-of-Fit Coefficient (GFC), and color difference (ΔE) values under D65 and A illuminants. The results revealed that reflectance measurements obtained at SPCA-selected wavelengths consistently yielded higher reconstruction accuracy than those derived from uniformly spaced sampling. Specifically, the SPCA-based measurements exhibited lower RMSE and ΔE and higher GFC values, demonstrating the superiority of data-driven wavelength selection under constraints of limited measurement channels.

The reflectance spectrum of an object represents its unique optical fingerprint, with each material exhibiting a distinct spectral signature that is crucial for object identification and colorimetric calculations^1–4^. Depending on the instrument’s capabilities and the intended application, reflectance spectra are discretely measured within the visible range using reflectance spectrophotometers or multispectral cameras. These instruments determine the percentage of spectral reflectance at various wavelengths relative to a standard white reference. Measurements are typically acquired from 400 to 700 nm, with wavelength intervals ranging from sub-nanometer to several tens of nanometers^2^. The number of measurement channels and their corresponding bandwidths directly influence the instrument’s ability to achieve accurate and meaningful results. For most natural and synthetic, non-fluorescent materials, the reflectance spectra are generally smooth across the visible range of the electromagnetic spectrum^4^. This smoothness allows for reliable color calculations even with relatively coarse wavelength sampling, ensuring consistent and accurate measurements for a wide range of applications^4^.

Depending on the instrument design, the commercial reflectance measurement spectrophotometers and multispectral cameras typically measure reflectance spectra in equal intervals within the visible spectrum, such as 5, 10–20 nm steps^3^. This uniform partitioning can overlook significant data if it does not account for the spectral behaviors of real objects. In other words, if the interval size used in spectral measurement is not adequately suited and is divided uniformly across the visible range, important spectral information may be missed, particularly when the bandwidths are not adequately optimized.

This study aims to identify the most informative sampling points within the visible region of the electromagnetic spectrum to minimize the number of measurement channels while reducing data loss as much as possible. To enable comparison with conventional spectrophotometers that employ uniformly spaced sampling intervals, the visible region was divided into equal steps of 20, 25, 30, 37.5, and 50 nm, corresponding to 16, 13, 11, nine, and seven sampling points, respectively. Sparse Principal Component Analysis (SPCA) was applied to the reflectance spectra of 1269 Munsell color chips measured at 1-nm intervals to determine the optimal sampling wavelengths. The resulting sparse eigenvectors were then used to identify the key wavelengths representing the most effective measurement points within the 400–700 nm range.

## Theoretical background

### From principal component analysis to its sparse variant

Various multivariate analysis methods have demonstrated that the reflectance spectra of objects exhibit strong correlations across the visible spectrum and can therefore be effectively represented by a reduced set of variables^5–14^. Among these methods, Principal Component Analysis (PCA) is the most widely used technique for dimensionality reduction in multivariate data^5–7,12−14^. However, a key limitation of PCA is that each Principal Component (PC) is expressed as a linear combination of all original variables, typically resulting in non-zero loadings for every variable. A simple approach to introduce sparsity, known as thresholding, sets the loadings of PCs to zero when their absolute values fall below a specified threshold. To address this issue more formally, Jolliffe et al. proposed the SCoTLASS algorithm, which incorporates a lasso-type penalty on the loadings within the PCA optimization framework^15^. Building on this concept, SPCA, introduced by Zou et al. in 2006, further mitigates the influence of all original variables on each PC, thereby overcoming one of the major limitations of conventional PCA^16^. Importantly, SPCA is designed to reveal meaningful patterns within high-dimensional datasets while simultaneously enforcing sparsity in their representation^17^.

Fundamentally, classical PCA can be formulated as a regression-type optimization problem which allows straightforward modification to incorporate sparse loadings. By introducing sparsity constraints into the optimization process, SPCA effectively captures the underlying structure of complex datasets using fewer non-zero coefficients, thereby enhancing interpretability in feature selection and data representation. Compared with conventional PCA, SPCA substantially reduces the number of variables required to describe a dataset by eliminating those associated with zero coefficients across all components. Furthermore, the use of sparse loadings facilitates efficient data storage and computation within sparse matrix structures, making SPCA particularly advantageous for high-dimensional spectral datasets or situations in which only a limited number of factors are influential. The primary limitation of SPCA, however, lies in its inability to preserve orthogonality among the components^16^.

A comprehensive overview of the theoretical framework underlying SPCA, including its mathematical formulation, optimization principles, and algorithmic implementations has been rigorously presented by Feng et al.^17^. Fundamentally, the classical PCA provides an optimal linear transformation that minimizes information loss while extracting a set of mutually uncorrelated orthogonal components^16^. The key objective of PCA is to identify directions (PCs) that capture the maximum possible variance of the original dataset. Mathematically, PCA seeks to project the covariance matrix X from the original *p*-dimensional feature space (**ℝ**^*p*^) onto a lower-dimensional subspace (**ℝ**^*q*^, where *q* ≤ *p*), while preserving as much of the total variance as possible.

This projection is obtained through a sequence of orthogonal transformations that ensure each PC is uncorrelated with the others. The mathematical formulation of PCA can be expressed as:

1 $$\:\underset{{\mathbf{v}}_{q}}{\max}~{\mathbf{v}}_{q}^{T}{\mathbf{X}}^{T}\mathbf{X}{\mathbf{v}}_{q},\:\text{subject to}\parallel\:{\mathbf{v}}_{q}{\parallel\:}_{2}=1$$

or equivalently, by minimizing the reconstruction error:

2 $$\:\underset{\mathbf{V}}{\min}\parallel\:\mathbf{X}-\mathbf{X}\mathbf{V}{\mathbf{V}}^{T}{\parallel\:}_{F}^{2}$$

where v_p_ denotes the *q*-th eigenvector of the covariance matrix X^T^X, representing the contribution (or loading) of each variable${\rm (x_1, x_2, . . ., x_p)}$to that PC. The matrix $V \in \mathbb{R^{\rm px \phi}}$comprises these eigenvectors as columns, and the corresponding eigenvalues quantify the amount of variance explained by each component. However, in conventional PCA, each PC typically involves all *p* variables with non-zero loadings. This full loading structure, while optimal for variance representation, often hampers interpretability and makes it difficult to identify the most influential variables.

To address this limitation, SPCA extends PCA by introducing sparsity into the loading vectors. The central idea of SPCA is to produce PCs with a limited number of non-zero coefficients, thereby facilitating variable selection and enhancing interpretability without substantially compromising explained variance. Conceptually, SPCA retains the variance-maximization objective of PCA but augments it with sparsity-inducing regularization constraints, typically via an L₁-norm penalty. The optimization problem can be expressed as:

3 $$\:\underset{{\mathbf{v}}_{q}}{\max}~{\mathbf{v}}_{q}^{T}{\mathbf{X}}^{T}\mathbf{X}{\mathbf{v}}_{q}-\lambda\:\parallel\:{\mathbf{v}}_{q}{\parallel\:}_{1},\text{subject to}\parallel\:{\mathbf{v}}_{q}{\parallel\:}_{2}=1$$

or equivalently, in its regression-based formulation:

4 $$\:\underset{\mathbf{V}}{\min}\parallel\:\mathbf{X}-\mathbf{X}\mathbf{V}{\mathbf{V}}^{T}{\parallel\:}_{F}^{2}+\lambda\:\sum\:_{q=1}^{Q}\parallel\:{\mathbf{v}}_{q}{\parallel\:}_{1}$$

where $\parallel \cdot \parallel_1$denotes the L₁-norm of the coefficient vector **v**, and **λ** is the sparsity-controlling regularization parameter, which may be common or component-specific. These formulations reveal the close relationship between PCA and regression-based regularization frameworks, in which the introduction of the lasso penalty (L₁ constraint) enforces sparsity in the loadings^16^. By zeroing out insignificant coefficients, SPCA enables the extraction of PCs that are not only representative of the data’s dominant variance structure but also interpretable in terms of the most relevant variables.

Given the structural and computational properties of SPCA, it can provide notable advantages over conventional PCA, particularly in terms of sparsity, interpretability, and variable selection capability. By enforcing sparsity in the loading vectors, SPCA effectively identifies a subset of variables (i.e., wavelengths) that contribute most significantly to the variance structure of the reflectance data. This sparsity constraint enables the method to isolate the most informative spectral bands while discarding redundant or highly correlated ones, thus yielding a compact yet representative feature set. Such selective dimensionality reduction is particularly beneficial when the number of available measurement channels is constrained, as in multispectral imaging or low-cost spectrophotometric systems. Consequently, SPCA not only enhances computational efficiency and interpretability but also improves the physical feasibility of measurement system design by guiding the optimal placement of spectral sampling points. In this context, SPCA serves as a data-driven wavelength selection framework that preserves the essential spectral characteristics of materials while minimizing the number of required measurements, thereby achieving an effective balance between accuracy, efficiency, and practicality.

### Data and method

In this study, SPCA was employed to identify the optimal sampling points for measuring the reflectance spectra of 1269 Munsell color samples. The spectral data were obtained from^18^, covering the 400–700 nm range at 1-nm intervals and were preprocessed using Savitzky–Golay smoothing filters (with a window length and the order of polynomial being 5 and 2 respectively) to reduce measurement noise^19^. To further evaluate the robustness and generalizability of the proposed wavelength selection method, an additional dataset comprising 175 reflectance spectra of oil-painted mock-up samples was also analyzed^20^.

The sequential variant of the SPCA algorithm proposed by Zou et al.¹⁶ was employed to compute the sparse PCs. This formulation was selected for its improved stability and interpretability, and it is available in a MATLAB toolbox that was used in this study. The sparsity of the loading vectors was controlled by the L₁ (lasso) penalty parameter, with sparsity levels of 16, 13, 11, nine, and seven examined to evaluate the effect of different numbers of nonzero loadings. In this procedure, all elements of each sparse eigenvector were constrained to zero except for those corresponding to the selected wavelengths, thereby identifying the most informative spectral positions for measurement. For comparison, an equivalent number of channels was also evaluated using uniformly spaced sampling points across the 400–700 nm range, corresponding to wavelength steps of 20, 30, 37.5, 50, and 60 nm for 16, 13, 11, nine, and seven channels, respectively. The selected and uniformly distributed wavelengths were subsequently assigned as the central positions of Gaussian filters with a Full Width at Half Maximum (FWHM) of one nm. Notably, an FWHM of one nm corresponds to approximately three non-zero spectral elements within each Gaussian channel, ensuring high spectral selectivity during reflectance reconstruction.

## Results and discussions

**Fig. 1:**
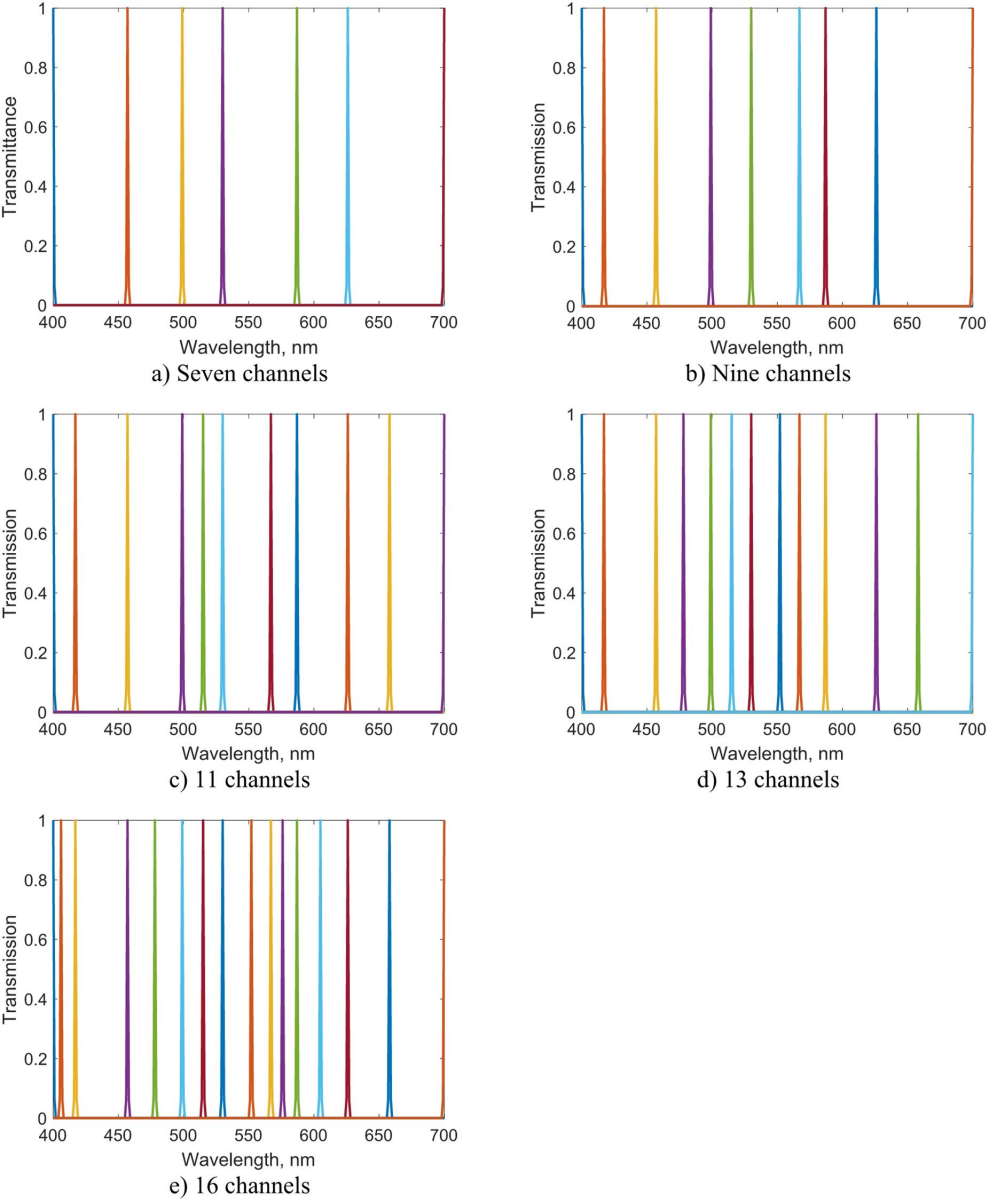
SPCA-selected spectral sampling points for seven, nine, 11, 13, and 16 measurement channels, implemented as the central wavelengths of Gaussian filters with uniform narrow bandwidth (FWHM = 1 nm). The nonuniform distribution of points reflects the spectral regions of greatest variation in the dataset.

Figure 1 illustrates the spectral positions of the measurement points selected by SPCA, shown as the central wavelengths of narrow-band Gaussian filters (FWHM = 1 nm). As observed, the selected sampling points are more sparsely distributed toward the longer-wavelength end of the visible spectrum and exhibit a denser clustering in the mid-visible region. This distribution suggests that the most significant spectral variations among the reflectance data occur in the mid-visible range, where the reflectance characteristics of materials change more rapidly, whereas relatively smaller spectral variations are present in the longer-wavelength region.

The reflectance spectra of the Munsell color samples were convolved with the desired Gaussian filters, and the resulting measurements were compared with those obtained from uniformly spaced sampling points using the same filter configuration. The spectral reflectances of the samples were subsequently reconstructed at one nm intervals from the sparsely sampled data using linear interpolation. The reconstruction performance of the two approaches, i.e. selective (SPCA-based) and uniform sampling, was quantitatively assessed by computing the Root Mean Square Error (RMSE) between the original and reconstructed spectra.

Table 1 summarizes the results in terms of the mean, maximum, and standard deviation of RMSE for different numbers of measurement channels. To further evaluate spectral similarity, the Goodness-of-Fit Coefficient (GFC) was also calculated, with the corresponding mean, minimum, and standard deviation values presented in Table 2. In addition, colorimetric accuracy was assessed using the CIE L*a*b* color-difference ${\rm (\Delta E_{ab}^*)}$ formula under the CIE 1964 standard observer and D65 and A illuminants, as summarized in Table 3.

**Table 1:** RMSE values between the original one nm resolution reflectance spectra and those reconstructed using Gaussian filters at wavelengths selected by SPCA or uniformly spaced across the visible spectrum.

# Channels	Method of selection	RMSE		
		Mean	Max	STD
7	SPCA	0.0115	0.0442	0.0095
	Equal	0.0122	0.0376	0.0089
9	SPCA	0.0063	0.0315	0.0046
	Equal	0.0091	0.0264	0.0062
11	SPCA	0.0043	0.0166	0.0029
	Equal	0.0061	0.0187	0.0043
13	SPCA	0.0032	0.0134	0.0020
	Equal	0.0043	0.0132	0.0030
16	SPCA	0.0022	0.0119	0.0013
	Equal	0.0028	0.0094	0.0017

**Fig. 2:**
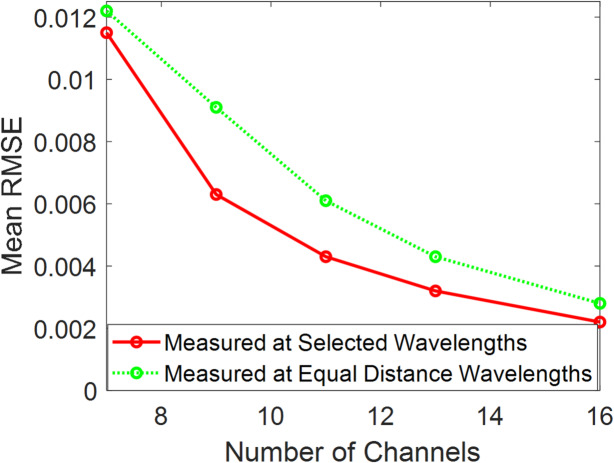
Effect of the number of measurement channels on the average RMSE for spectral reconstruction, comparing SPCA based selective sampling with uniform wavelength spacing.

The results in Table 1 indicate that SPCA-based wavelength selection outperforms uniform sampling, yielding consistently to lower mean RMSE values across all channel configurations. Figure 2 further shows that increasing the number of measurement channels reduces the average reconstruction error for both methods, emphasizing the trade-off between spectral sampling density and reconstruction accuracy.

**Table 2:** GFC values comparing the reference reflectance spectra (one nm resolution) with those reconstructed from SPCA based and uniformly spaced wavelength sampling.

# Channels	Method of selection	GFC		
		Mean	Min	STD
7	SPCA	0.9991	0.9675	0.0013
	Equal	0.9990	0.9623	0.0016
9	SPCA	0.9996	0.9753	0.0009
	Equal	0.9994	0.9910	0.0007
11	SPCA	0.9998	0.9777	0.0007
	Equal	0.9998	0.9923	0.0003
13	SPCA	0.9999	0.9981	0.0001
	Equal	0.9999	0.9979	0.0001
16	SPCA	0.9999	0.9981	0.0000
	Equal	0.9999	0.9995	0.0000

**Fig. 3:**
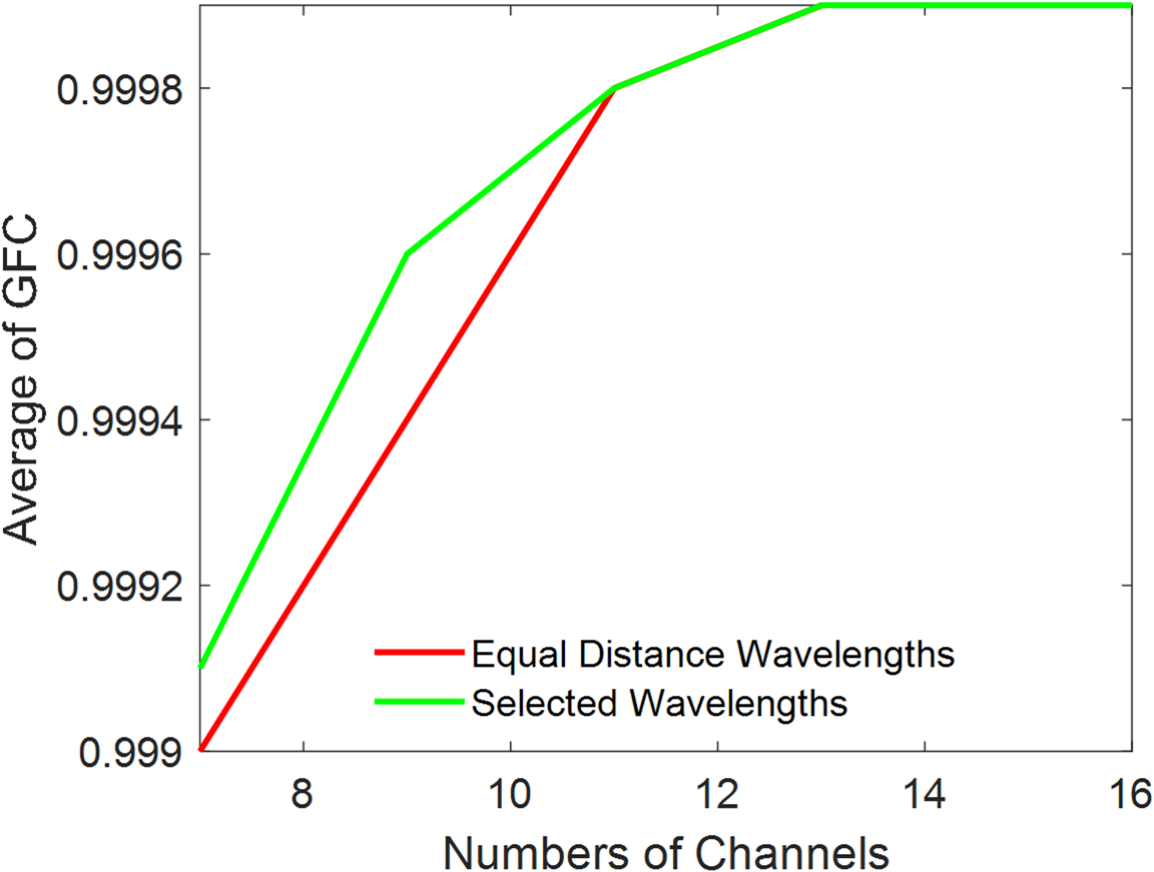
Effect of the number of measurement channels on the average GFC for spectral reconstruction, comparing SPCA-based selective sampling with uniform wavelength spacing.

Figure 3 illustrates that GFC improves as the number of measurement channels increases. Beyond 13 channels, the GFC values obtained from selective and uniform sampling become nearly identical, suggesting that both approaches achieve comparable reconstruction accuracy at higher channel densities.

The color difference values ${\rm (\Delta E_{ab}^*)}$ between the one nm reference spectra and those reconstructed from measurements at SPCA selected and uniformly spaced wavelengths were calculated under the CIE 1964 standard observer for D65 and A illuminants, with the results summarized in Table 3. The table indicates that SPCA based wavelength selection produces smaller mean ${\rm \Delta E_{ab}^*}$ values compared with uniform sampling when the number of channels is nine or fewer. However, this advantage diminishes as the number of measurement points increases, suggesting that the benefit of selective sampling is most pronounced at lower channel counts.

The significance of differences between the SPCA based selective and uniform (equal-distance) measurement methods was assessed using the non-parametric Wilcoxon signed-rank test, due to the non-normal distribution of the performance metrics. The results, summarized in Table 4, indicate that the selective-wavelength approach significantly outperformed uniform sampling across all evaluation metrics, including RMSE, GFC, and color-difference ${\rm (\Delta E_{ab}^*)}$  values under both D65 and A illuminants. These improvements demonstrate that selecting measurement wavelengths based on the spectral information content leads to more accurate spectral reconstructions and enhanced colorimetric fidelity.

In contrast, uniformly spaced sampling does not account for the nonuniform spectral variability often observed in reflectance data, particularly in regions exhibiting steep spectral gradients or pronounced absorption features. By allocating measurement channels to regions of greatest reflectance variation, the SPCA-based selective strategy provides a more efficient representation of spectral information, minimizing reconstruction errors. Consequently, this adaptive wavelength distribution improves the agreement between the one nm reference spectra and those reconstructed from sparsely sampled measurements, yielding lower RMSE and ${\rm \Delta E_{ab}^*}$ values and higher GFC coefficients. Overall, these findings confirm that the choice of measurement wavelengths is a critical determinant of spectral reconstruction accuracy, particularly when the number of available channels is limited.

**Fig. 4:**
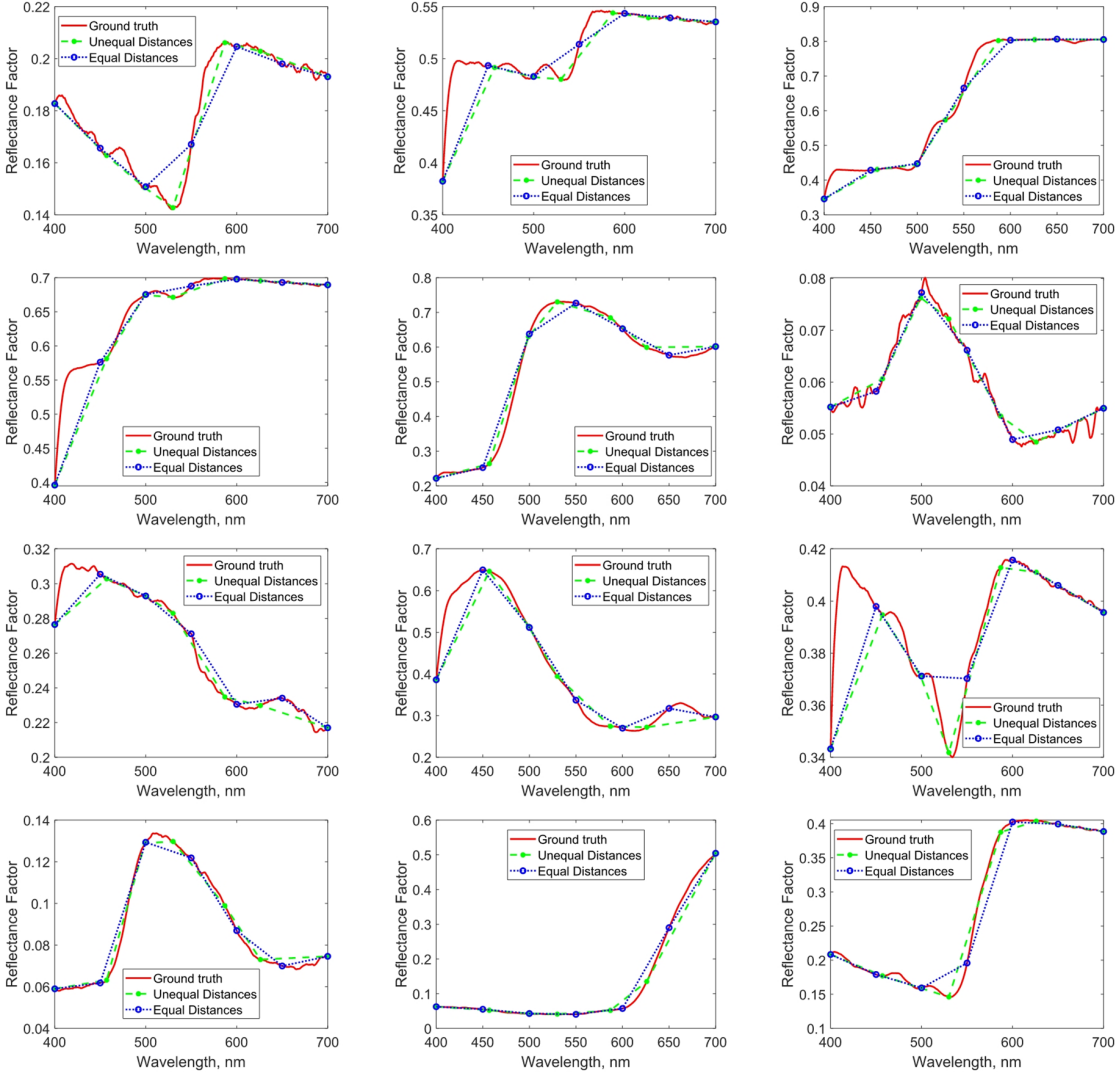
Comparison of the actual reflectance spectra and reconstructed spectra for 12 randomly selected samples. Reconstructions were obtained using selective (SPCA-based) and uniformly spaced sampling points with seven measurement channels.

**Fig. 5:**
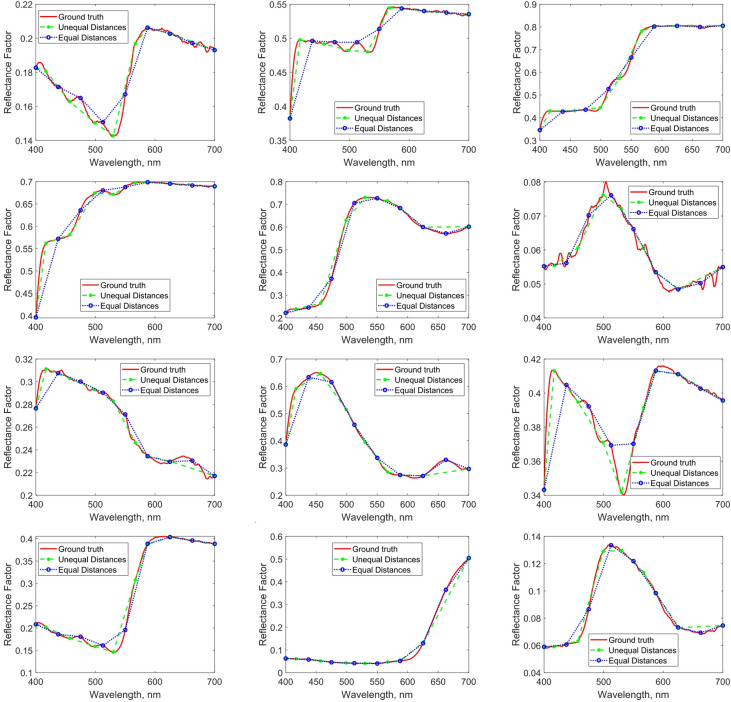
Comparison of the actual reflectance spectra and reconstructed spectra for 12 randomly selected samples. Reconstructions were obtained using selective (SPCA-based) and uniformly spaced sampling points with nine measurement channels.

**Fig. 6:**
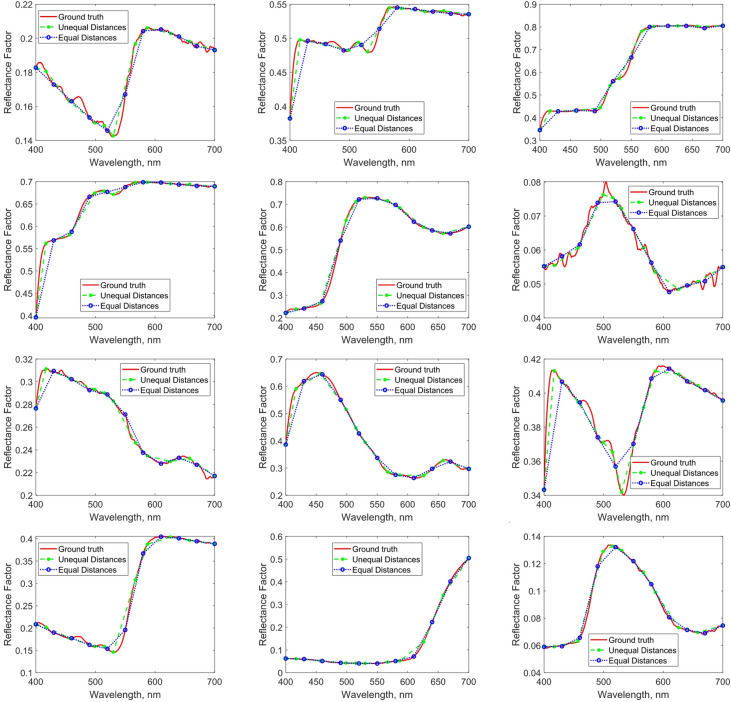
Comparison of the actual reflectance spectra and reconstructed spectra for 12 randomly selected samples. Reconstructions were obtained using selective (SPCA-based) and uniformly spaced sampling points with 11 measurement channels.

**Fig. 7:**
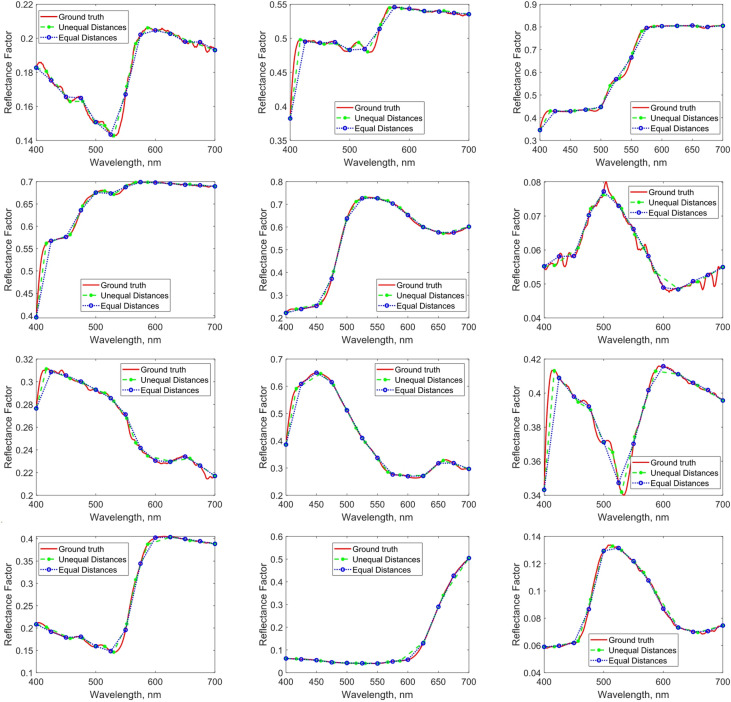
Comparison of the actual reflectance spectra and reconstructed spectra for 12 randomly selected samples. Reconstructions were obtained using selective (SPCA-based) and uniformly spaced sampling points with 13 measurement channels.

**Fig. 8:**
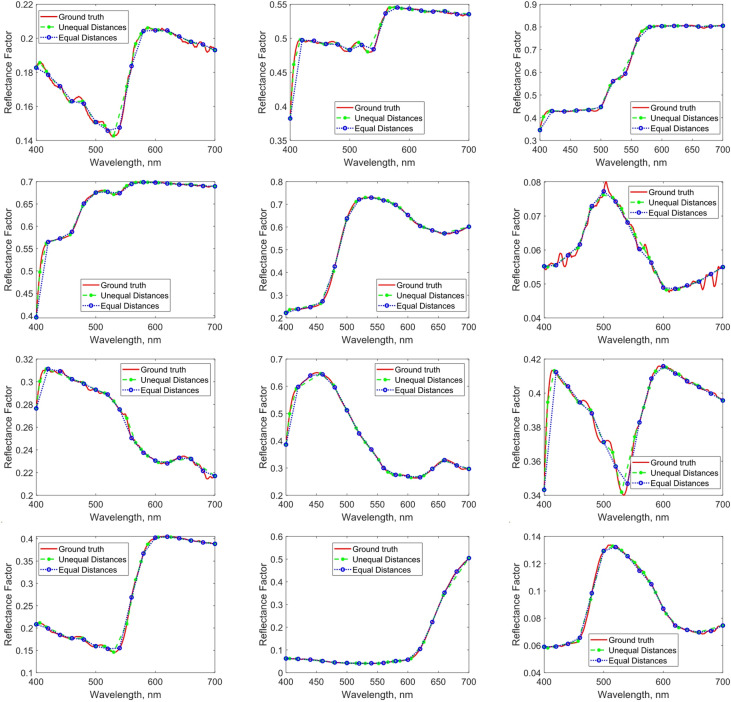
Comparison of the actual reflectance spectra and reconstructed spectra for 12 randomly selected samples. Reconstructions were obtained using selective (SPCA-based) and uniformly spaced sampling points with 16 measurement channels.

Figures 4, 5, 6, 7 and 8 present the measured reflectance spectra of 12 randomly selected samples alongside their corresponding reconstructions obtained from SPCA-based selective and uniformly spaced measurement points under varying numbers of channels. To ensure a fair and consistent comparison, the same set of randomly selected samples was used for all measurements across different channel configurations, allowing the effect of the number of channels on reconstruction accuracy to be clearly evaluated.

**Table 3:** ΔE* values comparing the one nm reference spectra with those reconstructed from selective (SPCA-based) and uniform wavelength sampling under the CIE 1964 standard observer for D65 and A illuminants.

# Channels	Method of selection	D65			A		
		Mean	Max	STD	Mean	Max	STD
7	SPCA	1.39	6.44	1.10	1.29	5.89	0.95
	Equal	1.749	6.89	1.24	1.58	7.03	1.18
9	SPCA	0.55	2.53	0.42	0.58	3.56	0.46
	Equal	1.09	3.95	0.71	0.88	3.35	0.58
11	SPCA	0.54	3.19	0.46	0.52	3.49	0.42
	Equal	0.46	1.80	0.29	0.40	2.06	0.27
13	SPCA	0.38	1.75	0.25	0.34	1.78	0.24
	Equal	0.23	0.90	0.14	0.20	1.06	0.12
16	SPCA	0.24	1.60	0.17	0.20	1.13	0.12
	Equal	0.16	0.97	0.11	0.16	0.96	0.11

**Table 4:** Statistical comparison of SPCA-selected and uniform wavelength sampling using the Wilcoxon signed-rank test. Performance differences were considered significant at *p* < 0.05.

# Channels	Parameters	RMS	GFC	ΔE	
				D65	A
7	Signed-rank statistics	366,837	475,961	275,933	285,437
	p-value	5.737 × 10^− 3^	2.209 × 10^− 8^	2.375 × 10^− 22^	2.332 × 10^− 19^
9	Signed-rank statistics	172,082	618,243	94,145	180,106
	p-value	6.23 × 10^− 70^	4.231 × 10^− 61^	1.284 × 10^− 123^	2.79 × 10^− 65^
11	Signed-rank statistics	186,702	584,078	442,959	527,382
	p-value	1.401 × 10^− 61^	8.995 × 10^− 44^	0.00216	1.53 × 10^− 21^
13	Signed-rank statistics	158,180	613,274	667,709	706,605
	p-value	2.235 × 10^− 78^	2.141 × 10^− 58^	1.942 × 10^− 9^	1.165 × 10^− 119^
16	Signed-rank statistics	190,625	594,659	604,679	512,979
	p-value	1.974 × 10^− 59^	8.018 × 10^− 49^	7.247 × 10^− 54^	3.463 × 10^− 17^

As evident from the figures, the spectral reflectance measurements obtained at SPCA-selected wavelengths exhibit a closer agreement with the one nm reference spectra compared with uniformly spaced sampling. This improved fidelity is particularly pronounced in regions of the spectrum with rapid reflectance variations or pronounced spectral features, where selective sampling captures the critical spectral information more effectively.

To further assess the robustness and generalization capability of the proposed wavelength selection methodology, an independent reflectance dataset was utilized^20^ that we call the 2nd dataset. Spectral measurements for 175 samples from this dataset were simulated using narrow-band Gaussian channel responses. The central wavelengths of these channels were determined either by the SPCA method or by a uniformly spaced wavelength configuration. Reconstruction performance was evaluated using three metrics, i.e. RMSE, GFC, and CIE color difference $(\Delta E^*_{ab})$ , under both D65 and A illuminants and the 1964 standard observer. A summary of the results is presented in Table 5.

The performance metrics exhibited variation not only with respect to the number of measurement channels but also in terms of their mean, maximum, and minimum values. In certain instances, the uniformly spaced configuration yielded marginally superior accuracy compared to the SPCA-based selection. This outcome is likely attributable to spectral bias within the employed dataset, which may disproportionately represent specific wavelength regions, thereby diminishing the efficacy of selective sampling strategies.

**Fig. 9:**
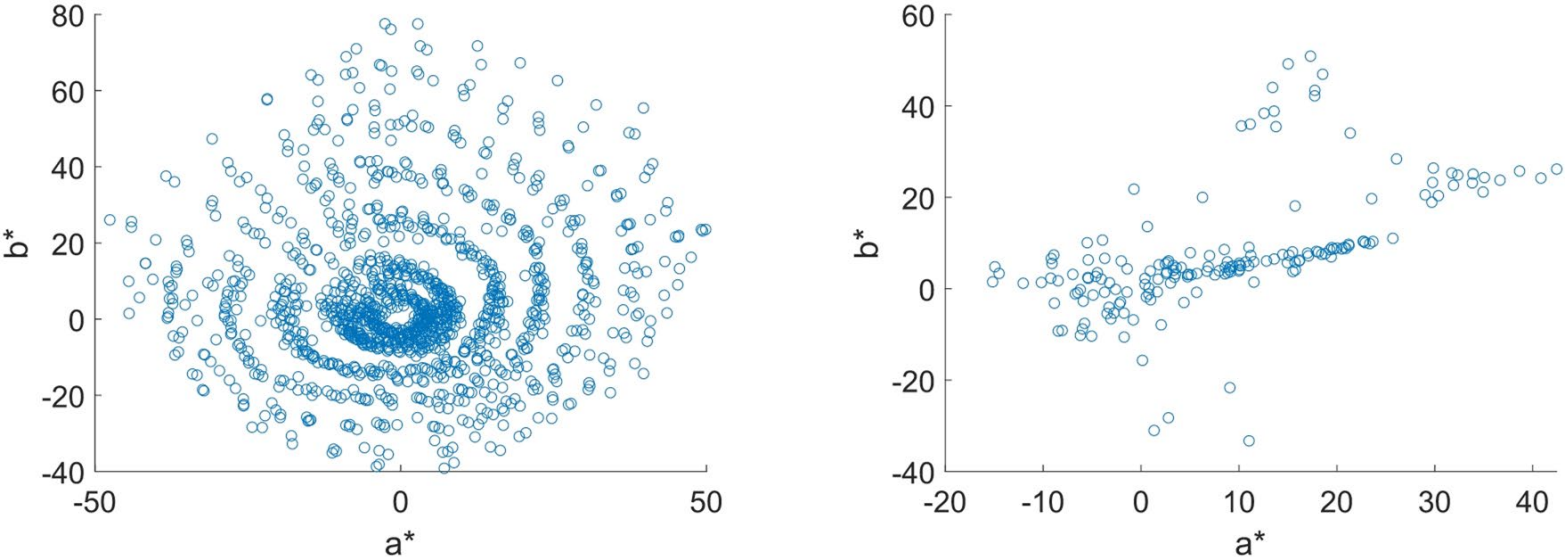
The a*b* distributions of samples of Munsell and test datasets.

To explore this problem from a colorimetric perspective, Fig. 9 illustrates the distribution of sample points in the CIELAB a*b* diagram, alongside Munsell reference samples, both computed under the D65 illuminant and the 1964 standard observer. Unlike the Munsell samples, which exhibit an approximately uniform distribution across the a*b* plane, the trial dataset demonstrates a non-uniform pattern, with samples clustering around specific hue regions.

**Fig. 10:**
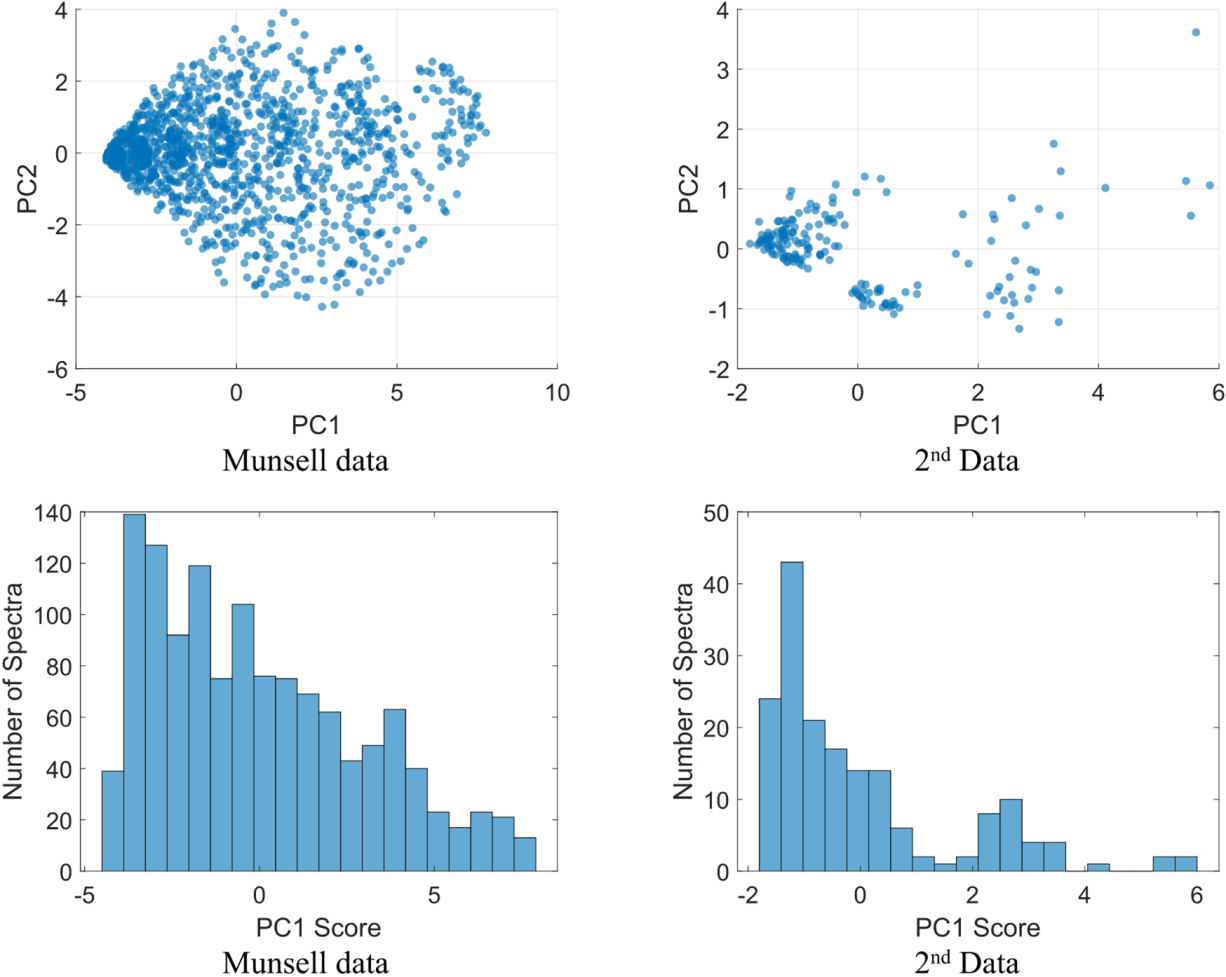
PCA scores for the Munsell and the test dataset, accompanied by histograms of the distributions along the first PC1.

To further characterize the spectral distributions, PCA was performed on both datasets. The loadings of the PCs represent the contribution of each original variable to a given component, indicating how strongly each variable influences the corresponding direction of maximum variance and providing valuable insight into the underlying structure of the data. The projections onto the first two PCs are shown in Fig. 10. The test dataset exhibits several localized clusters and a right-skewed distribution concentrated at lower PC1 values. The PC range for the Munsell dataset extends approximately from − 4 to + 8, whereas that of the test dataset is confined to a narrower range of -2 to + 6. This restricted and asymmetric spectral variance confirms the presence of sampling bias and redundancy, which may partly explain the discrepancies observed in reconstruction performance.

**Table 5:** Comparison of RMSE, GFC and ΔE values between the reference reflectance spectra of test dataset and those reconstructed using Gaussian filters with centers at optimal wavelengths (SPCA-based) and uniformly spaced wavelengths.

# Channels	Method of selection	RMS		GFC		ΔE			
						D65		A	
		Mean	Max	Mean	Min	Mean	Max	Mean	Max
7	SPCA Equal	0.0052	0.0174	0.9993	0.9952	0.93	2.88	0.85	2.91
		0.0046	0.0247	0.9994	0.9941	0.78	3.36	0.82	3.65
9	SPCA Equal	0.0038	0.0136	0.9996	0.9978	0.32	0.86	0.33	0.80
		0.0026	0.0107	0.9997	0.9887	0.44	3.00	0.42	2.36
11	SPCA	0.0020	0.0073	0.9999	0.9991	0.36	1.08	0.34	1.07
	Equal	0.0015	0.0078	0.9999	0.9991	0.21	0.75	0.19	0.77
13	SPCA	0.0017	0.0073	0.9999	0.9997	0.28	1.06	0.26	1.08
	Equal	0.0011	0.0050	1.0000	0.9992	0.10	0.51	0.10	0.41
16	SPCA	0.9482 × 10^− 3^	0.0035	1.0000	0.9999	0.06	0.17	0.06	0.15
	Equal	0.7233 × 10^− 3^	0.0028	1.0000	0.9997	0.06	0.36	0.05	0.20

## Conclusions

In this study, SPCA was employed to identify the most informative wavelengths across the visible spectrum using the reflectance data of 1269 Munsell color chips. The dimensionality of the spectral measurements was systematically reduced to 16, 13, 11, 9, and 7 channels within the 400 to 700 nm range. The selected wavelengths were implemented as the centers of Gaussian filters with a fixed FWHM of one nm to isolate the effect of wavelength choice. For comparison, an equivalent number of Gaussian filters with identical specifications but uniformly distributed central wavelengths were also applied, and the resulting reconstructions were benchmarked against the reference spectra.

Quantitative evaluation based on RMSE, GFC, and color difference (ΔE) under the 1964 standard observer with illuminants A and D65 consistently demonstrated the superiority of the SPCA-based wavelength selection over uniform sampling. Specifically, the SPCA-derived measurement sets achieved lower RMSE and ΔE values and higher GFC scores, confirming the effectiveness of data-driven wavelength optimization for compact spectral acquisition.

The robustness of the proposed approach was further validated using an independent dataset. Although the improvement in reconstruction metrics was less pronounced compared to the Munsell dataset used for SPCA implementation, the method still provided measurable advantages over uniform sampling. These findings highlight the potential of SPCA guided wavelength selection to enhance spectral measurement efficiency, particularly in applications constrained by limited measurement channels, and underscore its relevance for the design of compact, high-accuracy spectral imaging systems.

## Data Availability

The spectral data used in this study were obtained from the publicly available University of Eastern Finland dataset (https://sites.uef.fi/spectral/databases-software/munsell-colors-matt-spectrofotometer-measured/) and from Reference 20.
